# High Prevalence of Sickle Cell Disease in Low-Endemic Areas: A Pilot Study in Chunya Tanzania

**DOI:** 10.24248/eahrj.v9i1.835

**Published:** 2025-09-30

**Authors:** Amani Twaha, Deocles Donatus, Khanafi Said, Moshi Moshi Shabani, Marygladness Ngeme, Maryjesca Mafie, Stamily Ally Ramadhani, Abdulrahman Hussein, Nazareth M. Mbilinyi, Kasia Maksym

**Affiliations:** a Regency Medical Center, Ally Khan Road Dar es Salaam; b JK Hospital, Mikadi Beach Kigamboni, Dar es Salaam; c Kamextra Pharmacy, Chunya Tanzania; d The Research Department, One Health Society (OHS), Mpemba Street, Temeke, Dar es Salaam, Tanzania; e The Department of Biomedical Research and Clinical Trials, Ifakara Health Institute, Bagamoyo; f The Laboratory Services Department, Mbeya Zonal Referral Hospital; g University of Dar Es Salaam, Mbeya College of Health and Allied Sciences; h Department of Pediatrics, Mbeya Zonal Referral Hospital; i Elizabeth Gareth Anderson Institute for Women's Health, University College London; j Foetal Medicine Unit, University College London Hospitals NHS Trust

## Abstract

**Backround:**

Tanzania has the fifth highest prevalence of Sickle Cell Disease (SCD) worldwide. Annually, 11,000 children are born with SCD, but only 10% survive to their fifth birthday. Limited screening has led to underestimation of the burden in regions such as the southern highlands. The epidemiology of SCD, just like other diseases, is affected by climate change through increasing migration in search of arable land hence the shifts in the geographical prevalence of SCD from high prevalent areas to low prevalence areas. Early identification of SCD across all regions is therefore essential to improve survival, quality of life, mental health, reduce stigma, and alleviate the financial burden.

**Study Objective:**

The objective of the study was to assess the prevalence of SCD and SCT in Chunya district, Mbeya Region, Tanzania and to identify demographical factors associated with the risk of SCT among community members.

**Methods:**

A cross-sectional study on SCD was conducted in Chunya district, Mbeya Region, between 21^st^ and 22^nd^ February 2020. A total of 523 villagers were selected and screened for SCD and sickle cell trait (SCT) using rapid test (SICKLE SCAN(®).

**Results:**

The study revealed a notably high prevalence of SCD in southern highlands of Tanzania which highlighted the need for early screening and community-based awareness programs. The prevalence of SCD in the tested population was 1.91% and the prevalence of SCT was 8.41% of which the majority of the SCD patient were five years and below *P*=.02. Having a mother from Southern Zone was a protective factor (OR 0.2) against acquiring SCT while having a father from Northern Zone was a risk factor (OR 10), *P* value <.005.

**Conclusion:**

To reduce the burden of SCD, new strategies of screening should be developed to enable timely diagnosis and management of the disease.

## BACKGROUND

Hemoglobinopathies (Including Sickle Cell Disease) are among the most prevalent childhood Non communicable Diseases (NCDs) Worldwide. Other Important childhood NCDs include; nutritional disorders, cardiovascular diseases, paediatric cancers and chronic respiratory diseases^1^ treatments, and genetic counseling methods for sickle cell disease. This study was also devised to determine whether or not students, who are more likely to be genetically affected by sickle cell disease, are more or less aware of their sickle cell disease status. Two hundred and fifty-nine (259 Sickle Cell Disease (SCD) results from a single point mutation in the β-globulin gene (Glu6Val) leading to the production of haemoglobin S (HbS). Under deoxygenated conditions HbS polymerises, causing Red Blood Cells (RBCs) to sickle. The clinical manifestation of SCD are driven by recurrent vaso-oclusion, chronic haemolysis, systemic inflammation and progressive organ damage.^2^

The World Health Organization (WHO) estimated that over 5% of the world population carries genes associated with hemoglobinopathies. Worldwide, approximately 23 out of 10,000 people are affected by SCD, with the highest prevalence in African countries, where rates reach up to 110 per 10,000 people.^3^

Tanzania ranks fifth globally in the prevalence of SCD, with approximately 11,000 affected children born each year. Mortality rates can reach up to 90% in cases of suboptimal treatment.^4^

Patients with SCD benefit from a comprehensive and structured management approach, which includes prophylactic interventions against common co-morbidities such as bacterial infections and organ dysfunction. Regular screening for disease related complication such as cerebrovascular events and ocular manifestations is crucial, Early detection and timely intervention significantly reduce morbidity, enhance quality of life, and increase life expectancy in individuals living with SCD.

Chronic diseases have shown to trap families into a spiral of poverty and disease, suggesting that SCD may be a significant driver of sustained poverty in Tanzania. Despite this heavy burden, policy responses to SCD in Tanzania remain limited, with unmet need for investment in diagnostic facilities and modern therapeutic interventions.^2^ For instance, recent advancements in the management of SCD through genomics, bone marrow transplants and novel pharmacological therapies aimed at improving the quality of life of patients with SCD^5^ have yet to be widely accessible in developing countries like Tanzania.

Given the anticipated increase in children born with SCD in sub-Saharan Africa and Tanzania, it is crucial to identify sickle cell patients and provide timely, comprehensive treatment. In Tanzania, sickle cell centres are located in high-endemic regions such as Dar Es Salaam, Mwanza and Zanzibar.^6^ In low-prevalence areas like Kilimanjaro and Mbeya, services are integrated into paediatric or internal medicine departments.^6,7^ However, the pattern of sickle cell prevalence has shifted due to climate change and population dynamics, with migration introducing the disease to previously low-endemic areas.^8^

Sickle cell centres offer high-quality clinical and laboratory services and maintain a sickle cell patients registry. However, in presumed low-endemic areas such as Mbeya, data on prevalence is scarce due to limited screening, low community awareness, and long distances many villages are from care facilities. Moreover, cultural practices such as consanguineous marriage may further contribute to unexpectedly high prevalence rates in these regions ^9^.

Therefore, the study aims to assess opportunities for improving the quality of care for individuals living with Sickle Cell Disease in Chunya, Mbeya, Tanzania.

## METHODS

### Study Design

A cross-sectional survey was conducted in Chunya District in February 2020. Any individual residing in Chunya were eligible to participate. Adults aged 18 years and above who provided written consent to were included in the study. For participants under 18 years of age, assent was obtained from the child in addition to written consent from a parent or legal guardian.

### Data Collection Team

This project was inherently multidisciplinary. To effectively serve the primary beneficiaries (Rural Communities in Southern Tanzania), the study team comprised a paediatrician, nurses, lab scientists and an epidemiologist. Medical student volunteers also participated in data collection.

Prior to fieldwork, the team received training on Good Clinical Practices (GCP) and data collection ethics. The aim was to minimise bias and ensure Reliability and validity of the data.

### Data Collection

#### Phase I: Training of the medical student volunteers and quality control.

Medical student volunteers were trained by paediatricians, laboratory scientists and haematologists on the fundamentals of SCD. Stakeholder meetings were held to disseminate plans for testing and to secure support for transportation and equipment. Quality control procedures were conducted to verify the reliability of the testing kits prior to community wide mass screening.

#### Phase II: Community mobilization

Following the training, an awareness campaign was conducted through visits to secondary school, hospitals, religious gatherings, market places and community centres to inform residents about the planned screening activity and blood testing for Sickle Cell Trait (SCT) and SCD. In addition to in-person visits, conventional and social media campaigns were conducted reaching out to approximately 20,000 individuals. Selection bias was minimized through multiple recruitment sites, and data collection points across the district, including hard-to-reach communities.

#### Phase III: Data Collection

Participants were identified using a simple random selection approach among volunteers. Demographic data was collected using questionnaires. Participants were asked to provide information on their ethnic and demographic, status, knowledge, and attitude towards SCD. For laboratory assessment of SCT and SCD, Point of Care test was performed using Sickle Scan Rapid test devices manufactured by BioMed omics.^10^

Sickle SCAN is a multiplexed, qualitative, point-of-care immunoassay designed for rapid diagnosis of sickle cell disorders. The test employs three indicators to detect the presence of haemoglobin A, S and C, allowing the user to rapidly distinguish between normal, carrier and SCD samples. The essay has demonstrated 100% sensitivity and specificity for AA Controls and HbCC cases, with a specificity of 99.2% overall.^11^ Moreover, it is capable of detecting SCD in individuals as younger as one day old.^10^ All results were recorded electronically using questionnaires and securely deposited in REDCap®.^12^

#### Phase IV: Geospatial analysis on the prevalence of SCD and SCT

Geospatial analysis of Sickle Cell Disease (SCD) was conducted using QGIS software.^13^ These studies provided a framework for analysing the spatial distribution of SCD prevalence. We measured the prevalence rates in regions previously identified as high endemic areas, ensuring consistency and comparability with historical data. By mapping these prevalence, we were able to visualise the geographic distribution and identify regions with higher rates, such as Mbeya. This mapping facilitated a deeper understanding of regional variations and potential factors contributing to the increased prevalence in specific areas.

### Data Validity and Reliability

The study ensures data validity and reliability through endeavours such as having data managers at each data collection site to ensure quality data collection, Data was double entered in the data collection software and later cleaned by the head data manager.

Prior to data collection, all data collectors were trained on data collection methods and GCP.^14^ Further-more, data quality was supervised by the University of Dar es Salaam and Mbeya Zonal Referral Hospital.

### Data Analysis

Collected data was analysed using STATA version 13.^15^ Key variables were summarised using frequencies and percentages (for binary or categorical variables) and mean and standard deviation (for continuous variables). Bivariate logistic regression models were then constructed to assess associations between potential risk factors and the presence of SCT and SCD. Chi-squared tests and Adjusted Odds Ratio (AOR) were calculated to quantify the strength of these associations.

In the bivariate logistic regression models, we examined risk factors such as the presence of a sibling with SCD and the geographic origin of the family. Variables that showed statistical significance in the bivariate analysis were included in the multivariate models. These multivariate models were adjusted for all significant risk factors identified in the bivariable analysis. This approach allowed us to explore and account for potential confounding factors and to better understand the relationship between these risk factors and the presence of SCT.

Potential Confounders: Geographic location, health-seeking behaviour, and access to health services were considered potential confounders, given their possible association with both the exposures (e.g., parental origin) and the outcomes (SCD/SCT status). These variables were adjusted for in the multivariable logistic regression model.

**Primary Outcomes:** The main outcomes were the presence of SCD and SCT, as determined by the Sickle Scan® point-of-care test.

**Predictors:** Predictor variables included sociodemographic factors such as; age, sex, parental origin (Southern Highlands, Lake Zone, Coastal Zone), and family history of SCD (defined as having a sibling or parent with SCD or SCT).

**Potential Confounders:** Geographic location, health-seeking behaviour, and access to health services were considered potential confounders due to their possible association with both the exposure (e.g., family origin) and the outcome (SCD/SCT status). These variables were adjusted for in the multivariable logistic regression model.

**Effect Modifiers:** The role of family history and parental origin was also assessed as potential effect modifiers, especially in the interaction between geographic background and SCD prevalence. Where appropriate, stratified analyses were conducted.

### Ethics Approval

Ethical approval was obtained from the University of Dar es Salaam Ethics Clearance Sub-Committee (Approval No. AB 458/482/02/412). All members of the study steering committee declared no conflicts of interest prior to data collection.

The investigators confirm that the study was conducted in accordance with the Principles of the Declaration of Helsinki, as amended in Tokyo (1975), Venice (1983), Hong Kong (1989), South Africa (1996), Edinburgh (2000), Washington DC (2002), Tokyo (2004), Seoul (2008) and Fortaleza (2013) and adopted by the World Medical Association in 2022.^28^ The study also adhered to the Tanzanian Ministry of Heath guidelines.

All members of the study steering committee declared no conflict of interest prior to data collection.

Informed consent was obtained through clear communication between guardians and the research team, using Swahili or local languages. The study's purpose, methods, risks, and benefits were explained respectfully, ensuring voluntary participation without coercion. Confidentiality was emphasized, and participants retained the right to withdraw at any time. All procedures complied with ethical regulations, upholding individual rights and dignity.

The study team assumed day-to-day responsibility for ensuring compliance with Good clinical practice (GCP) requirements, including quality control, quality assurance of the data collected as well as safety reporting. The Principal Investigator maintained close communication with individual sites and study supervisors to ensure that these processes were effective implemented.

## RESULTS

### Data Summary

**FIGURE 1:**
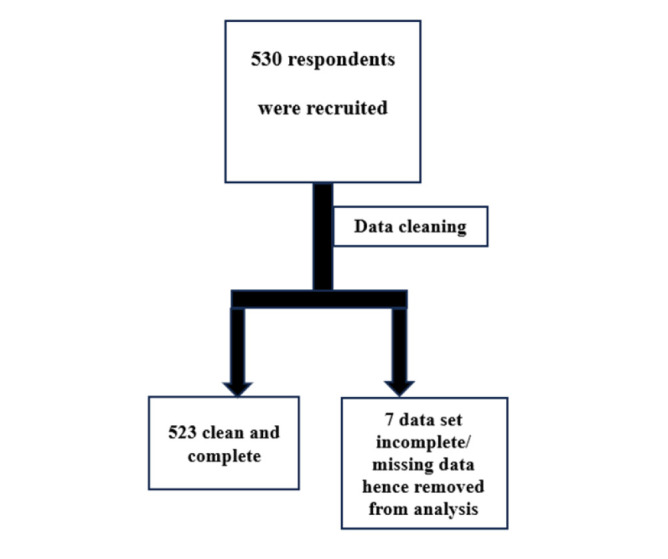
Participant Inclusion and Exclusion Summary

A total of 530 participants were recruited into the study. The complete data set was available for 523 participants and this cohort was analysed further. (Figure 1)

### Demographic Characteristics

Among the respondents, 53.92% were female and 46.08% were male. The respondent's age ranged from 1 day up to 64 years, with a median age of 16 years, and Standard Deviation (SD) of 9.8. The majority of respondents reported that both their father and mother were illiterate (52.2% and 60.04%, respectively). Participants reported belonging to 49 different ethnic groups, which for the purposes of analysis were divided into 4 zones based on their geographical location. These zones were: Southern Zone, Central Zone, East and Northern Zone (Table 1).

**TABLE 1:** Demographic Characteristics of the Respondents

Variable	Frequency	Percentage
Sex		
Male	241	46.08
Female	282	53.92
Parents’ level of education		
Father		
Did not attend formal education	273	52.2
Primary School	106	20.27
Secondary School	16	3.06
Advanced Secondary School	15	2.87
College	37	7.07
University & higher learning Institutions	76	14.53
Mother		
Did not attend formal education	314	60.04
Primary School	87	16.63
Secondary School	6	1.15
Advanced Secondary School	18	3.44
College	19	3.63
University & higher learning Institutions	79	15.11
Parents’ zonal tribes Father		
Father		
Southern zone	423	80.88
Central Zone	48	9.18
Northern & Eastern Zones	52	9.94
Mother		
Southern zone	421	80.5
Central Zone	54	10.33
Northern & Eastern Zones	48	9.18

### The Prevalence of Sickle Cell Disease in Mbeya Tanzania

The prevalence of SCD was 1.91% among the 523 tested community members. Most cases (70%) were identified in children aged five years and below (*P* = .02). (Table 3)

### The Prevalence of Hemoglobinopathies and Sickle Cell Traits

**FIGURE 2:**
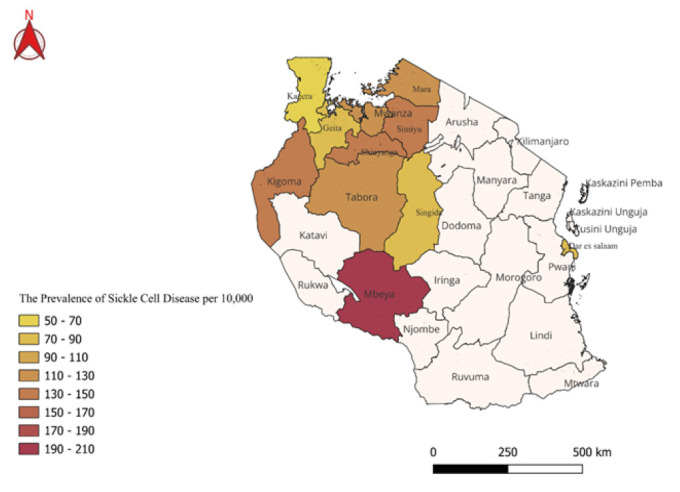
Map of Tanzania Showing Distribution Pattern and Prevalence of Sickle Cell Disease.

Among the 523 individuals, 89.7% had normal haemoglobin (AA), 8.4% were carriers (HbAS), 0.25% had HbSC and 1.7% had Sickle Cell disease (HbSS) (Figure 2).

### Demographic Risk Factors for Sickle Cell Trait

Having a sibling with SCD was strongly associated with an increased likelihood of carrying SCT, with the risk estimated to be nearly 11 times higher (AOR= 10.8, *P=.02*).

**FIGURE 3:**
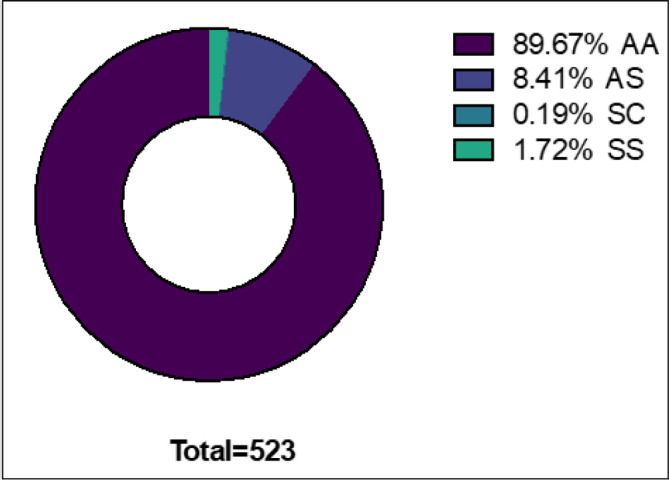
Prevalence of Different types of Hemoglobin Amongst 523 respondents

Respondents whose maternal tribe originated from Southern zone ethnic groups was found to be protective, reducing the risk of SCT by approximately 80% (*P= .004*). (Figure 3) In contrast, paternal origin from the Lake zone ethnic groups was associated with a tenfold higher risk of SCT compared to paternal origin from other regions (*P=.012*) (Table 2).

**TABLE 2:** Adjusted Odds Ratio for the Demographic Risk Factors to Acquire Sickle Cell Disease

SCD risk	Odds Ratio	Standard Error	Z Score	*P value*	95% Confidence Interval	
					95% Lower	95% Upper
SCD in family						
Sibling	10.75	10.50	2.43	.02	1.58	72.91
None	1.83	0.93	1.19	.23	0.67	4.98
Mothers’zonal tribe						
Lake and Coastal Zone	0.81	0.61	−0.28	.78	0.18	3.50
Southern Highlands	0.19	0.12	−2.84	.00	0.06	0.60
Father’zonal tribe						
Lake and Coastal Zone	10.06	9.23	2.52	.01	1.66	60.75
Southern Highlands	4.86	3.75	2.05	.04	1.07	22.04
cons	0.03	0.03	−3.77	.00	0.00	0.20

**TABLE 3:** Relationship Between Age Category and Sickle Cell Disease

Age Category	Sickle Cell Disease status (n)		
	Negative	Positive	Total
Infants (0–1)	64	2	66
Young Children (1–5)	46	3	49
Children (5–12)	18	2	20
Adolescents (12–18)	239	2	241
Young Adults (18–30)	120	1	121
Middle-aged Adults (30–45)	20	0	20
Older Adults (45+)	6	0	6
Total	513	10	523

### Geospatial Analysis

**FIGURE 4:**
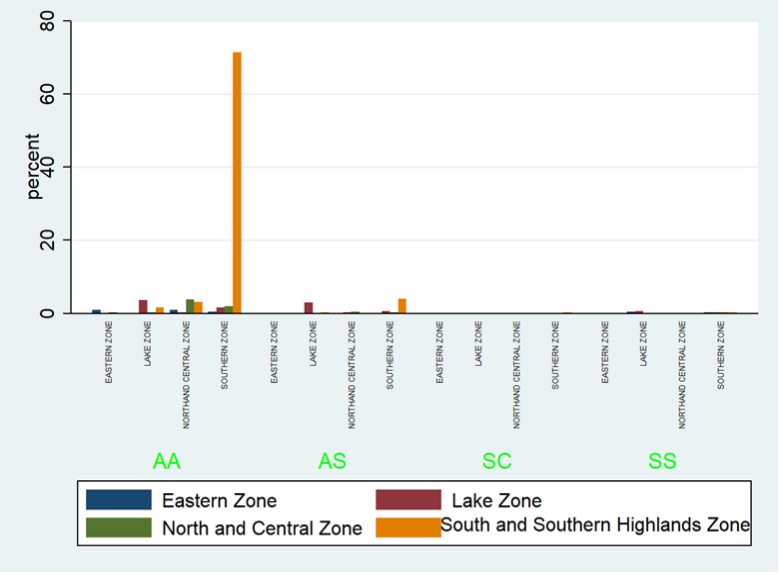
Bar Graph Distribution of Different Hemoglobinopathies Across the Zones

Geospatial mapping was conducted to visualize the distribution of SCD and SCT cases across Chunya District and regions of Tanzania with known past prevalence. (Figure 4) Geographic coordinates from screening sites were collected and analysed using QGIS software. These were integrated with historical prevalence data to identify spatial patterns and potential high-risk zones.

## DISCUSSION

### Summary of the Key Findings

Among 523 respondents, 53.92% were female and 46.08% were male. Participants’ age ranged from 1 day to 64 years, with a median age of 16 years (SD = 9.8). The study included individuals from 49 ethnic groups, which were categorised into four major zones based on geographical position; Southern Highland, Central, lake and Eastern zones.

The prevalence of SCD was 1.91%, while the prevalence of SCT was 8.4%. Notably, 70% of individuals with SCD were aged five years and below.

There was a significant relationship (*P*=.00) between SCT and parental geographical origin. Those whose maternal side originated from Southern highlands were 80% more protected against acquiring SCT, whereas those whose paternal side originated from Lake and Eastern Zones where 10 times more likely to acquire SCT.

### Implications of the Study Findings

The observed associations between geographic or ethnic background and SCD/SCT prevalence are not intended to stigmatize any group, but rather to highlight the shifting epidemiology of SCD due to migration and demographic dynamics. These findings support the need for universal screening policies that are inclusive and responsive to population changes, aiming to promote equity in early diagnosis and care across all regions.

In this study, the prevalence of SCD among 523 individual was observed to be 1.91%. This prevalence is higher than global, regional, and national estimates. The World Health Organization (WHO) estimates the global prevalence of SCD at 0.23%^3^, with over 5% of the population carrying genes associated with hemoglobinopathies. In Africa, the prevalence of SCD is approximately 1.1%.^16^ Locally, studies in Tanzania report varying rates: one study in in Dar Es Salaam reported a prevalence of 0.8%^17^, while another study conducted in Bugando reported prevalence of 1.2.^18^

The high prevalence of SCD observed in our study is likely multifactorial. Regional variations in the distribution of hemoglobinopathies could contribute to these discrepancies. Genetic predisposition within certain populations, coupled with environmental factors may also play contribute significantly to the elevated prevalence in our study area.

One significant hypothesis is that migration into the study area, which previously had a lower prevalence of SCD, has contributed to the increased disease risk. The influx of individuals from regions with higher SCD prevalence (Especially Lake zone) in search of arable land, may have contribute to this increase. Migration impacts genetic landscape in a population and this can potentially lead to higher rates of hereditary conditions such as SCD.^19^ Understanding migration patterns and the origins of the new residents could provide valuable insights into the genetic composition of the current population and the factors influencing SCD prevalence.

Secondly, the study population's demographic characteristics may have influenced the findings. Factors such as age distribution and ethnicity could impact the prevalence of SCD. It is possible that our sample population had a higher proportion of individuals from ethnic groups with a known predisposition to SCD.

Furthermore, methodological differences across studies may also account for variations in reported prevalence. Differences in diagnostic criteria, screening methods, and study designs can significantly influence the variations in reported prevalence rates.^20^ Our study employed a comprehensive screening and diagnostic approach, which may have identified cases that were overlooked in earlier studies.

The higher prevalence of SCD observed in this study highlights the importance of targeted public health interventions and policies tailored to specific regional needs. Strengthening screening programs, promoting early diagnosis, and expanding access to comprehensive healthcare services for individuals with SCD are essential to reduce disease burden and improve health outcomes in affected communities.

In this study, the prevalence of SCT was 8.4% among 523 respondents. This is notably lower than the prevalence reported in Bugando Hospital, where the percentage of sickle cell carriers was found to be 20.3%.^21^ Understanding the carrier status, particularly among individuals of reproductive age is essential for healthcare preparedness, as it enables early interventions and adequate planning for the management of potential Sickle cell newborns.^9^ Several factors may explain the observed discrepancy. Geographic and population-based genetic variations are significant considerations; different regions may have varying carrier rates due to historical and demographic factors. It is possible that the population in our study area has a lower SCT prevalence due to lower baseline gene frequencies or less historical exposure to the trait compared to the population around Bugando Hospital.

Furthermore, understanding the carrier status of individuals of reproductive age helps in genetic counselling and family planning, enabling couples to make informed decisions regarding childbearing and the prevention of potential risks of SCD in their offspring.^22^ Awareness of SCT prevalence is equally important for public health planning, as it ensures that healthcare services are adequately prepared to manage and treat newborns with SCD.^9^ Early diagnosis and timely interventions play a pivotal role in improving survival, reducing complications, and enhancing the overall quality of life and outcomes for children born with SCD.^23^

Furthermore, the knowledge of SCT prevalence in different regions can guide targeted public health interventions, such as education campaigns, screening programs, and resource allocation. Suc efforts are critical in mitigating the impact of SCD by facilitating early diagnosis, strengthening preventive strategies, and ensuring the provision of comprehensive care for affected individuals.^24^

The study assessed risk factors associated with acquiring SCT and found that having a sibling with SCD increased the risk of acquiring SCT by 11 fold. Conversely, maternal lineage from Southern Zone ethnic group was found to be protective against SCT, reducing the risk by approximately 80%. On the other hand, having a father whose tribe originates from the Northern and Central Zones, increased the risk of SCT 10 folds. These findings correlate to the malaria adaptation theory and the natural history of sickle cell disease, in which populations living near the equator, particularly in Africa, exhibit higher carrier rates due to evolutionary adaptations against Plasmodium Falciparum.^25^ Moreover, the genetic inheritance pattern of SCD supports the observed association, as individuals with affected sibling inherently carry a higher risk of inheriting SCT.^26^

These findings align with the genetic inheritance patterns of SCD, where the likelihood of inheriting the trait is markedly higher among individuals with affected family members due to the autosomal recessive nature of the disease. This underscores the importance of family history in assessing and predicting the risk of SCT. Furthermore, such information is particularly valuable to health care workers in remote areas with limited access to diagnostic tools, as it can enhance clinical suspicion and targeted screening. Recognising these factors may therefore promote increased vigilance in the identification of individuals at risk and facilitate appropriate linkage to care and genetic counselling.

Moreover, the natural history of SCD, characterised by the genetic inheritance of the sickle cell gene, supports our findings. The risk of inheriting SCT is strongly influenced by parental carrier status.^17^ The elevated risk observed among individuals with fathers from Northern and Central Zone tribes may reflect historical genetic patterns and the varying prevalence of the sickle cell gene in different regions of the country.

This study highlights the complex interplay of genetic, familial, and regional factors in the inheritance of SCT. These insights are crucial for developing tailored public health strategies to address the burden of SCD and improve health outcomes for affected populations. Nevertheless, further research is needed to explore the underlying genetic mechanisms and validate these findings.

Additionally, the study highlighted an important relationship between age category and SCD, where by approximately 70% of SCD positive individuals were within the 0 to 5 years age category. This finding echoes well-documented evidence that majority of children with SCD in sub-Saharan Africa do not survive to see their 5th birthday.^23,27^ This underscores the urgent need for enhanced efforts in early diagnosis, comprehensive care and effective linkage to healthcare services to reduce mortality and morbidity associated with SCD in early childhood.

### Strength of the Study

This multidisciplinary study was strengthened by strong community engagement and support, including volunteer labour from medical students, the donation of testing kits, and active participation from the local communities.

The use of point of care diagnostics further enhanced the study, as these tools are affordable, easy to use and practical for deployment in resource limited settings.

Importantly, this is the first study conducted in this geographical region, providing valuable pilot date and insight into the burden of SCD and SCT.

The study has provided predicting factors that can aid health care workers to increase index of suspicion to diagnose SCD and SCT which is beneficial in limited scarce environments.

### Study Limitations

This study had several limitations that should be acknowledged. First, selection bias may have occurred because participants were recruited through a community outreach campaign rather than random sampling. Although efforts were made to include diverse populations across various geographic and demographic settings in Chunya District, the non-randomised recruitment limits the generalizability of the findings to the entire district or country.

Second, there is a possibility of information bias, especially related to self-reported variables such as family history of SCD or place of origin, which may be prone to recall error or misclassification. However, the use of structured interviews and trained data collectors helped to reduce this risk.

Third, residual confounding cannot be ruled out. While multivariable logistic regression was employed to adjust for key factors, unmeasured variables such as socioeconomic status, health literacy, and access to healthcare services may have influenced both the likelihood of participating in the screening and the observed outcomes.

Fourth, the cross-sectional design limits causal inference. The observed associations between predictors and SCD/SCT prevalence indicate correlation but cannot establish temporal or causal relationships.

Despite these limitations, the study provides valuable insights into the changing epidemiology of SCD in a region historically considered low-prevalence. It also highlights the feasibility and impact of youth-led, community-based screening approaches in resource-limited settings. Furthermore, the integration of geospatial analysis strengthened the contextual understanding of disease distribution and migration-related patterns of risk.

## CONCLUSION

There is a high prevalence of SCD, as well as a high risk of SCT transmission in Mbeya influenced by multiple factors including migration patterns and geographical location.

The findings of this study are not intended to stigmatise or discriminate against any ethnic group or geographical location, rather, they aim to enhance awareness and increase the index of suspicion among healthcare workers when assessing individuals at risk. By identifying these predictive factors, the study contributes to improved screening, early diagnosis, and appropriate linkage to care in resource-limited settings.

### Recommendation

This study represents a novel pilot investigation in the Southern Highlands of Tanzania. Given that many regions of the country remain unexplored, there is a clear need for larger, population-based studies to provide more accurate estimates of the national prevalence of SCD in the country. Particular focus should be placed on the most remote areas, with the introduction of comprehensive Neonatal Screening programmes to enable early identification and timely linkage to care. Additionally, there is a pressing need to strengthen existing healthcare infrastructure by equipping a dedicated Sickle Cell Unit at Mbeya Zonal Referral Hospital and transforming it into a fully functional sickle cell centre.
